# Change in the Prevalence of Testing for Latent Tuberculosis Infection in the United States: 1999–2012

**DOI:** 10.1155/2016/1850879

**Published:** 2016-05-30

**Authors:** Nicholas T. Vozoris, Jane Batt

**Affiliations:** ^1^Tuberculosis Program, Division of Respirology, Department of Medicine, St. Michael's Hospital, Toronto, ON, Canada M5B 1W8; ^2^Li Ka Shing Knowledge Institute, St. Michael's Hospital, Toronto, ON, Canada M5B 1W8; ^3^Department of Medicine, University of Toronto, Toronto, ON, Canada M5G 2C4

## Abstract

*Purpose*. There is no information on the change in prevalence of latent tuberculosis infection (LTBI) testing in the United States (US) following the introduction of the interferon gamma release assay (IGRA), a new and alternative diagnostic method for LTBI. The purpose of this study was to evaluate potential changes in the prevalence of LTBI testing in the US following the introduction of IGRA.* Methods*. This was a multiyear cross-sectional study using nationally representative data from the 1999-2000 and 2011-2012 US National Health and Nutrition Examination Surveys. Self-reported prevalence of LTBI testing was estimated among groups known to have increased LTBI risk. Descriptive statistics were used.* Results*. Compared to 1999-2000, significantly fewer individuals self-reported being tested for LTBI in 2011-2012 among Hispanic Americans (68.0% versus 60.7%, *p* < 0.0001) and among those with comorbidities (74.7% versus 72.0%, *p* = 0.02). There were also nonsignificant trends towards less self-reported LTBI testing in 2011-2012 versus 1999-2000 among household contacts of active TB cases, foreign-born individuals, and African Americans.* Conclusions*. Despite the introduction of IGRA, LTBI testing occurs less frequently in the US among vulnerable groups. Possibly inadequate targeted LTBI testing could result in increased active TB in the US in the future.

## 1. Introduction

In 1989, the United States (US) Centers for Diseases Control and Prevention set an aggressive national target of eliminating active* Mycobacterium tuberculosis* (TB) disease as a public health problem [1]. To reach this goal, identification and prophylaxis of individuals with latent TB infection (LTBI) at increased risk for progression to active disease are required and it is recognized that these strategies are likely being underutilized in the US [1]. In 2011-2012, about 5% (13 million individuals) were estimated to have LTBI in the US [2], which is up from 4% (11 million people) in 1999-2000 [3, 4]. Sociodemographic groups in the US that have been identified as having increased risk for LTBI include African Americans, Hispanic Americans, foreign-born individuals, and household contacts of active TB cases [3, 4]. Individuals within these groups would be at increased risk for developing active TB if they were either recently infected or if they had certain clinical conditions associated with progression from latent to active disease [5, 6].

Tuberculin skin testing (TST) is the traditional tool for identifying LTBI. There are several limitations associated with TST: there are potential inaccuracies with test implantation and reading; two visits are required, one for test implantation and a second one for reading 48–72 hours later; and false positives can occur as a result of nontuberculous mycobacterial infection or previous Bacille Calmette-Guerin (BCG) vaccination, because the TST material contains antigens found in most nontuberculous mycobacterium strains and BCG [7]. In 2001, an alternative method for LTBI testing was introduced in the US, the interferon gamma release assay (IGRA) [7]. The IGRA is a blood test that measures the amount of interferon gamma released from T cells upon exposure to TB-specific peptides [7]. The original IGRA test introduced in the US in 2001, QuantiFERON-TB Test, was withdrawn from the market in 2005 because of poorer specificity relative to TST [7]. Three IGRA products are currently available in the US: QuantiFERON-TB Gold Test (QFT-G) (available since 2005); QuantiFERON-TB Gold In-Tube Test (QFT-GIT) (available since October 2007); and T-Spot (available since July 2008) [7]. Compared to TST, the currently available IGRA products offer advantages of greater specificity (e.g., 99% for QFT-GIT versus 85% for TST) with similar sensitivity [7], increased convenience as only a one-time blood draw is required, and fewer inaccuracies with test administration. However, the IGRA is more costly and it may not be readily available to all individuals. Other considerations relating the IGRA include limited performance data for children < 5 years and for immunocompromised individuals [7] and great variation in results when serial testing is performed [8]. Patient groups in whom IGRA testing is preferable over TST include those that historically have poor return rates for TST reading (e.g., the homeless population) and individuals who have previously received BCG vaccine [7].

In 1999-2000, prior to the introduction of the IGRA, about 35% of foreign-born individuals and 13% of household contacts of active TB cases in the US were estimated to never have received LTBI testing [4]. One might anticipate that the frequency of testing in such LTBI-vulnerable groups would have improved following the introduction of a second LTBI diagnostic test, associated with greater convenience and specificity. There is some evidence indicating that the availability of IGRA improves medical compliance: receipt of LTBI chemoprophylaxis is greater among those tested by the IGRA versus TST [9]. By extension then, the IGRA may also improve patient adherence to LTBI testing. There is no information available on the change in prevalence of LTBI testing in the US since the introduction of the IGRA. The purpose of this study was to evaluate potential changes in the prevalence of LTBI testing in the US, following emergence of the IGRA, among selected vulnerable groups.

## 2. Methods

This was a multiyear cross-sectional study using US nationally representative population-level data from the National Health and Nutrition Examination Survey (NHANES). The NHANES is a cross-sectional survey that is conducted in the US every two years and each survey sample represents the total noninstitutionalized civilian US population residing in the 50 states and District of Columbia. Sociodemographic and health information is collected from participants in person by trained professionals. Information from survey participants under 16 years of age is collected with the assistance of a parent or guardian being present.

Questions related to previous testing for LTBI were included on the 1999-2000 and 2011-2012 survey cycles (Table 1). In 1999-2000, survey participants ages ≥ 1 years were asked whether or not they had ever had a TST or a tine test. In 2011-2012, participants aged ≥6 years were asked whether or not they had ever had an IGRA, TST, or tine test. As part of the LTBI test questioning in both survey cycles, a brief description of TST and tine testing was provided to participants, and for the 2011-2012 survey cycle pictures of what a TST and tine test look like were also shown to participants. Individuals who refused to respond to questions about LTBI testing or responded “do not know” were excluded from this analysis (4.3% of 1999-2000 sample; 4% of 2011-2012 sample). Individuals with a known or uncertain history of self-reported doctor-diagnosed active TB were also excluded from this analysis (1% of 1999-2000 sample; 1% of 2011-2012 sample). The reason for excluding the latter group is that individuals with previous active TB would have no need for LTBI testing.

Testing for LTBI was evaluated among five preselected groups of individuals: household contacts of active TB cases; foreign-born individuals; African Americans; Hispanic Americans; and individuals with certain comorbidities (specifically, asthma, chronic obstructive pulmonary disease [COPD], arthritis, cancer, and diabetes). Information regarding these variables was collected from survey participants via self-report and comorbidities were self-reported doctor-diagnosed conditions. The first four selected groups were chosen by the authors because they have been previously identified as being at increased risk for LTBI [3, 4]. Comorbidities of asthma, COPD, arthritis, and cancers were selected by the authors because receipt of immunosuppressive medications (like systemic corticosteroids) that are used as treatment in these diseases has been independently associated with increased risk of developing active TB [10, 11]. Furthermore, diabetes [12], cancers (especially nonsolid tumours) [13], and smoke exposure [14] (which is the most common cause of COPD [15]) have been independently associated with progression to active TB. We acknowledge that not all individuals within our five selected groups would require LTBI testing. However, if testing was found to be occurring less frequently over time within these vulnerable groups, this would be potentially concerning, because individuals within these groups with LTBI at increased risk for progression to active TB may not be identified and may not be offered chemoprophylaxis.

The prevalence of self-reported LTBI testing was calculated for each of our five selected groups in 1999-2000 and in 2011-2012. If an individual who had received LTBI testing belonged to more than one than LTBI risk factor group, he or she was included in the prevalence estimate for each group. Chi square test of proportions was used to determine statistically significant differences in prevalence estimates between the two time periods using *p* < 0.05 threshold. NHANES uses a complex sampling design, employing stratification and multistage clustering [16]. To account for the unequal probabilities of selecting respondents, all point estimates were appropriately weighted using the survey sample weights provided [17]. All analyses were performed on SAS version 9.4. Ethics approval was granted by the St. Michael's Hospital Research Ethics Board.

## 3. Results

In the 1999-2000 NHANES cycle, the numbers (and percentages) of individuals in our five selected groups who did not previously have active TB and who responded to the question relating to LTBI testing were as follows: household contact with an active case of TB = 223 (2.7%); foreign-born individuals = 1190 (14.6%), African Americans = 978 (12%), Hispanic Americans = 1309 (16%); and individuals with selected comorbidities = 2580 (41.6%). In the 2011-2012 NHANES cycle, the corresponding numbers (and percentages) were as follows: household contact with an active case of TB = 207 (2.4%); foreign-born individuals = 1393 (16.1%); African Americans = 1072 (12.4%); Hispanic Americans = 1375 (15.9%); and individuals with selected comorbidities = 3255 (45.7%).

**Figure 1:**
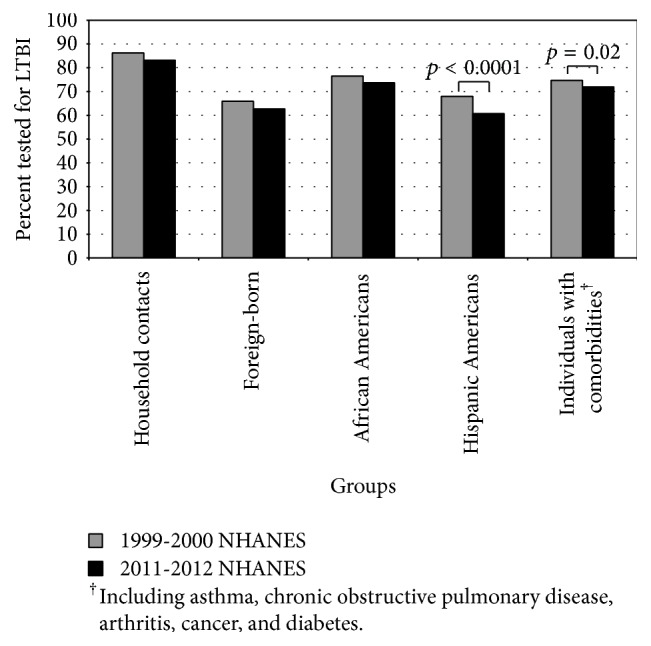
Change in the self-reported prevalence of latent TB infection (LTBI) testing in the United States among vulnerable groups, 1999-2000 versus 2011-2012 National Health and Nutrition Examination Surveys (NHANES).

Compared to 1999-2000, significantly fewer individuals self-reported testing for LTBI in 2011-2012 among Hispanic Americans (68.0% versus 60.7%, *p* < 0.0001) and among those with comorbidities (74.7% versus 72.0%, *p* = 0.02) (Figure 1). There were also nonsignificant trends towards less self-reported LTBI testing in 2011-2012 versus 1999-2000 among household contacts of active TB cases, foreign-born individuals, and African Americans.

## 4. Discussion

To our knowledge, this is the first study to describe changes in the self-reported prevalence of LTBI testing in the US over time. There were trends towards lower self-reported prevalence of LTBI testing in 2011-2012 versus 1999-2000 among multiple vulnerable groups and significantly lower prevalence among Hispanic Americans and individuals with comorbidities. This occurred despite introduction into the market of an alternative diagnostic tool to the TST, the IGRA, in 2001 [7]. Our finding of decreasing LTBI testing among multiple at-risk groups is particularly concerning, given that LTBI has slightly increased over the same time period in the US [2].

The decrease in LTBI testing prevalence between 1999-2000 and 2011-2012 may be underestimated given that a smaller proportion of younger individuals were asked about LTBI testing in 2011-2012 compared to 1999-2000 (i.e., LTBI screening was asked of those ≥1 years in 1999-2000 versus those ≥6 years in 2011-2012). Analysis of 1999-2000 NHANES data shows that younger children are less frequently tested for LTBI than older children (LTBI testing prevalence is 47.3% in children 1–5 years versus 50.9% in children 6–12 years). The decrease in LTBI testing prevalence between 1999-2000 and 2011-2012 may also be underestimated given that pictures of what TST and tine testing look like were shown to participants as part of the 2011-2012 cycle, but not as part of the 1999-2000 cycle. Had such images been shown to participants in the 1999-2000 cycle, this may have led to the finding of a higher reported LTBI testing prevalence for that time.

The finding of lower self-reported LTBI testing frequency in 2011-2012 may be in part explained by changes in LTBI testing strategies that have occurred in the US over time. Historically, all US residents were recommended to have at least one TST in their lifetime [18]. However, more recent US TB guidelines in 2000 recommended focusing testing towards individuals at risk for LTBI with increased risk with diseased activation [5]. However, there have been no policy changes regarding use of BCG vaccination in the US during the time period examined to contribute to the lower prevalence of LTBI testing in 2011-2012 [5]. Although financial cost and availability may have limited use of IGRA testing, the less expensive and more readily available TST remained an option for use in 2011-2012. Indeed, the majority of those tested in the US by 2011-2012 received TST (78%). It is possible that the decline in self-reported LTBI testing reflects a perception among patients and health care providers that targeted testing is less relevant or necessary to be performed because of the decreasing trends in active TB cases in the US over recent decades [19].

The finding of lower prevalence of LTBI testing over time consistently across multiple vulnerable groups raises concern that targeted LTBI testing is actually occurring less frequently. Should targeted LTBI testing be inadequate, this could result in increased active TB in the US in the future, especially since LTBI has slightly increased over time in the US [2] and since the proportions of foreign-born individuals and individuals with comorbidities among the total US population are increasing. Although our estimates of LTBI testing in 1999-2000 among foreign-born individuals (65.9%) and household contact of active TB cases (86.2%) were slightly different from that previously reported by others using the same data set (foreign-born individuals = 65.2%; household contacts of active cases = 87.2% [4]), these discrepancies can be explained by methodological differences (i.e., the latter estimates were based on data from participants receiving a TST during NHANES and individuals with any missing data were recoded and not excluded [4]).

There are several study limitations. Our estimates of LTBI testing could be overestimated as a result of participants misinterpreting the LTBI testing question with BCG vaccine receipt. This potential limitation likely applies only to foreign-born individuals, as awareness and receipt of BCG vaccine would likely apply only to this group. Alternatively, LTBI testing could be underestimated as a result of recall bias or lack of awareness by some individuals that they had been previously tested by a simple blood draw. Results may have also been influenced by the difference in wording and content of the LTBI testing questions between the two survey cycles. For example, images of TST and tine testing were not made available in the 1999-2000 cycle, and while TST and tine testing were described to participants in both survey cycle questions, a description of the IGRA was not provided in the 2011-2012 cycle. Although data on LTBI testing in this study was based on patient self-report, which potentially introduces recall bias, objective measures of LTBI testing are not easily available and evaluation of previous LTBI testing in the “real clinical world” is based on patient self-report. NHANES did not collect data relating to reasons for LTBI nontesting and further research would be required to identify the factors contributing to the trends in decreasing LTBI testing in the US. The five selected groups at risk for LTBI that we studied also reflect a mixture of different and uneven LTBI risk factors. Finally, information was not available in NHANES to allow for evaluation of LTBI testing in other relevant vulnerable groups, such as individuals with HIV, organ transplantation, end-stage renal failure on hemodialysis, and recipients of immunosuppressive medications (like antitumour necrosis factor agents).

## 5. Conclusions

Despite the introduction of a new and alternative testing method for LTBI, there were trends towards less LTBI testing over time in multiple vulnerable groups in the US and particularly Hispanic Americans and individuals with comorbidities. These findings raise concern that targeted LTBI testing may actually be occurring less frequently over time in the US. Possibly inadequate LTBI testing in the US may impede attaining the aggressive national TB elimination goals that have been set there.

## Figures and Tables

**Table 1:** Latent TB infection (LTBI) testing questions contained in the 1999-2000 and 2011-2012 National Health and Nutrition Examination Surveys (NHANES).

NHANES cycle	LTBI testing question
1999-2000	“Have you ever been given a TB or tuberculosis skin test? For one version of this test, a doctor or nurse presses a plastic button with little metal prongs down your arm. That kind is called a tine test. For another version of this test, they use a small shot needle to stick a few drops of tuberculin or PPD just under the skin.”

2011-2012	“The next questions are about being tested for tuberculosis. The tests could be a skin test with a needle just under your skin, a blood test, or a plastic button with metal prongs pressed on your arm called a tine test. Here are pictures of what the skin test and tine test look like. Have you ever been tested for TB? Which test or tests did you receive, the needle under the skin, the blood test, or the tine test?”
